# Effects of Chemical Compositions on Plating Characteristics of Alkaline Non-Cyanide Electrogalvanized Coatings

**DOI:** 10.3390/nano10112101

**Published:** 2020-10-23

**Authors:** Thanyalux Wanotayan, Pongsakorn Kantichaimongkol, Viriyah Chobaomsup, Sirikarn Sattawitchayapit, Klaus Schmid, Martin Metzner, Tongjai Chookajorn, Yuttanant Boonyongmaneerat

**Affiliations:** 1 Metallurgy and Materials Science Research Institute, Chulalongkorn University, Pathumwan, Bangkok 10330, Thailand; thanyalux.w@chula.ac.th (T.W.); viriyah.ch@student.chula.ac.th (V.C.); 2 Nanoscience and Technology Interdisciplinary Program, Graduate School, Chulalongkorn University, Pathumwan, Bangkok 10330, Thailand; 6288308020@student.chula.ac.th; 3 National Metal and Materials Technology Center (MTEC), National Science and Technology Development Agency (NSTDA), Pathum Thani 12120, Thailand; sirikars@mtec.or.th; 4 Department of Electroplating, Fraunhofer Institute of Manufacturing Engineering and Automation IPA, Stuttgart 70569, Germany; klaus.schmid@ipa.fraunhofer.de (K.S.); martin.metzner@ipa.fraunhofer.de (M.M.)

**Keywords:** electrodeposition, zinc, nanostructures, characterization

## Abstract

The effects of zinc and sodium hydroxide concentrations in an alkaline non-cyanide zinc bath on the electrodeposition characteristics of zinc deposits are systematically investigated. Using microstructural and phase analyses of specimens with specifically designed geometries, the study indicates that the bath formulations critically control the electrogalvanizing characteristics and affect the coating surface morphology, deposition rate, throwing power, coating uniformity, and residual stresses developed during and after electrogalvanizing. The coatings produced from baths with a moderate Zn-to-NaOH ratio of 0.067–0.092 appear to provide uniform and compact deposits, moderately high deposition rate, and relatively low residual stresses.

## 1. Introduction

Owing to their relatively simple fabrication process and ten to hundred times higher corrosion resistance than ferrous materials depending on the environment, electrogalvanized zinc coatings have been widely employed to protect steels from corrosion in many industries ranging from automotive and machine elements to construction [1,2,3]. Among the plating electrolyte systems available, including the acid-based and alkaline-based baths [4,5,6], the alkaline non-cyanide zinc bath has gained increasing interest due to its simple bath composition (merely two main chemicals), low internal stress, and low tendency for hydrogen embrittlement (less than 1 ppm critical hydrogen concentration) [7,8,9]. Nevertheless, fundamental studies aiming to understand the influencing factors of plating quality and to establish a process–property relationship of electrogalvanized coatings plated from the alkaline non-cyanide bath have been very limited.

Several process parameters and variables such as chemical compositions of plating baths can potentially play a critical role in influencing the characteristics of electrogalvanized coatings, including the plating coverage, throwing power, and uniformity of coating thickness [10,11]. Moreover, these factors could also affect the development of internal stresses, which could subsequently result in the failure of deposited films, by mechanisms such as blistering or delamination. The effects of process variables on residual stress and how it, in turn, affects the properties of the deposits have been examined, primarily focusing on copper and nickel films [12,13,14,15]. However, the study on electrodeposited zinc is very limited and has been largely focused on the acid bath and the effects of organic additives [16,17,18,19,20]. Among various process parameters, plating temperature, pH of plating baths, and current density are found to influence the morphology and corrosion behavior of zinc deposits [21,22,23,24,25].

In this work, the effects of zinc and sodium hydroxide concentrations on the electrodeposition characteristics of electrogalvanized zinc coatings of the alkaline non-cyanide system are systematically investigated. Particularly, deposition rate, thickness uniformity, and internal stresses developed during and after plating are analyzed with respect to the bath composition. The understanding gained from this study provides fundamental knowledge for further development of the electroplated zinc coatings and also benefits practical uses and controls of the electrogalvanized plating system.

## 2. Materials and Methods 

### 2.1. Electrodeposition Setup

Zinc layers were electrodeposited onto low-carbon steel substrates using a 5 L alkaline non-cyanide zinc bath. The zinc electrolytes were prepared by dissolving zinc oxide (ZnO) powder and sodium hydroxide (NaOH) pellets (Carlo Erba Reagents, Bangkok, Thailand) in deionized water. The concentrations of Zn and NaOH were varied systematically for different baths, to be termed A–G, as listed in Table 1. Practically, for the alkaline non-cyanide zinc bath, the normal operating range for Zn was 8–11 g/L, and for NaOH, it was 120–150 g/L. The bath composition was designed herein to study the effect of both Zn and NaOH concentrations within the operating range and also when they are out of range from both ends. Cationic polyamines-based, aldehyde-based, and thiourea-based additives (Columbia Chemical, Bangkok, Thailand) were added into the bath with a corresponding concentration of 15, 0.25, and 1 mL/L to produce coatings with good appearance and uniform thickness. Prior to electrodeposition, the substrates were pretreated by soak cleaning in 50% NaOH at 70 °C for 3 min, electro-cleaning in 5% NaOH at 5 V for 30 s, and pickling in 5% HCl (Carlo Erba Reagents, Bangkok, Thailand) for 10 s. The electrodeposition was conducted with a current density of 2 A/dm^2^ for 30 min at room temperature, using low-carbon steel anodes surrounding the specimens. 

### 2.2. Plating Characteristics

#### 2.2.1. Plating Uniformity Assessment

**Figure 1:**
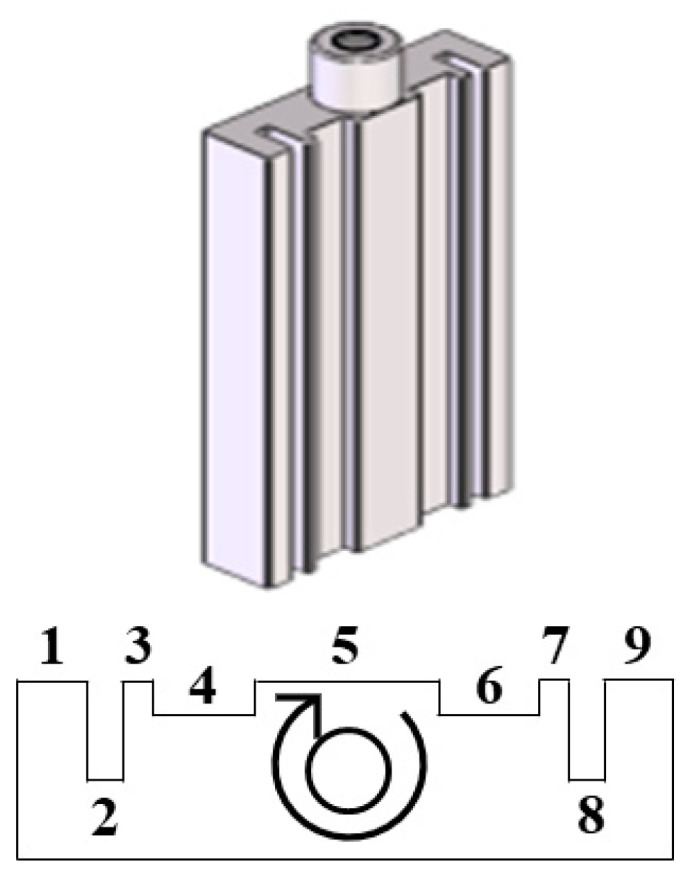
Corrugated specimen as a rotating cathode.

The plating uniformity assessment is designed to realistically simulate typical industrial working conditions, in which work pieces are inevitably exposed to nonuniform electric fields and bath agitation. By introducing these effects in a controlled manner, this assessment can therefore be used to examine the throwing power and hydrodynamic effects in a plating session. Figure 1 shows a specially designed corrugated specimen with outer dimensions of 80 mm × 38 mm × 10 mm and a total surface area of 1 dm^2^. The corrugated specimens are divided into nine zones with two different groove depths that experience different local current densities. Thus, the throwing power, which is the ability to apply coating with a uniform thickness over an irregularly shaped cathode, can be determined by measuring the coating thickness inside the grooves with respect to that on the outside surface. During the plating process, the specimen also rotates in a clockwise direction at 100 rpm (Figure 1). This results in a variation in fluid velocities in different zones.

#### 2.2.2. Structural Examination

Optical microscopy (OM) was used to determine the coating thickness from a cross-sectional view of the corrugated specimens. Scanning electron microscopy (SEM, JSM-IT500HR, JEOL Ltd., Tokyo, Japan) was employed to analyze the surface morphology at zone 5 of the electrogalvanized corrugated samples. The phase identification was carried out at the same zone using X-ray diffractometry (XRD, PW3710, Philips, Almelo, Netherlands) with a Cu radiation source and a scan rate of 0.02 °/min.

### 2.3. Stress Measurements

#### 2.3.1. In-Situ Stress Measurement 

Steel test strips with a size of 8 mm × 180 mm × 50 µm were used for the in-situ (during plating) stress measurement and the post-plating stress measurement of deposits from the baths listed in Table 1. The internal stress of the electrodeposits was determined using the change of length method. The strip was aligned by fixing one end and attaching the other end to a dial gauge. During the electroplating process, the length of the strip would change in response to the stress build-up inside the test strip. The reading from the gauge was then converted to the stress of the deposits, such that the internal stress of the zinc coating, *σ* (MPa), was estimated using Equation (1),

(1) $$\sigma =-\frac{E_{m}t_{m}+2E_{p}t_{p}}{2l_{p}t_{p}\left(1-\nu \right)}\times 1000\times \Delta_{m}$$

where *E_m_* and *t_m_* are respectively the elastic modulus (185 GPa) and thickness (50 µm) of the measuring strip, *E_p_* and *t_p_* are the elastic modulus (200 GPa) and thickness of the plating, respectively, *l_p_* is the plated length of the measuring strip (180 mm), *ν* is Poisson’s ratio (0.3), and *Δ_m_* is the signed length change of the measuring strip, with a negative value indicating an increase in the strip length. From each bath, three specimens were electroplated, and an average value is used to represent the internal stress obtained during electroplating.

#### 2.3.2. Ex-situ Stress Measurement

Following the electrodeposition and the in-situ stress measurement, surface residual stress measurements were performed on the electroplated test strips using a µ-X360s X-ray residual stress analyzer (Pulstec Industrial Co.,Ltd., Hamamatsu, Japan) with Cr radiation. A single incident angle X-ray exposure, or in other words, the cos α method, was performed by analyzing a Debye ring from the diffraction cone associated with the Zn (112) plane using an incidence angle of 35° and an irradiation time of 30 s. The residual stress was then estimated based on the distortion of the Debye ring in stressed specimens using the Young’s modulus of 200 GPa and the Poisson’s ratio of 0.3. Three specimens from each bath were measured and an average value was used to represent the surface residual stress resulting from each bath formulation.

## 3. Results and Discussion

**Figure 2:**
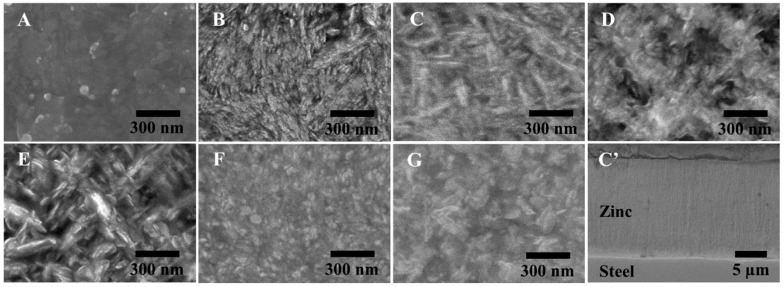
Scanning electron micrographs of electroplated zinc surface with different Zn/NaOH ratios: (**A**) 0.042, (**B**) 0.067, (**C**) 0.092, (**D**) 0.117, (**E**) 0.122, (**F**) 0.073, (**G**) 0.061, and (**C’**) a representative cross-sectional view of the coating prepared from bath C.

**Figure 3:**
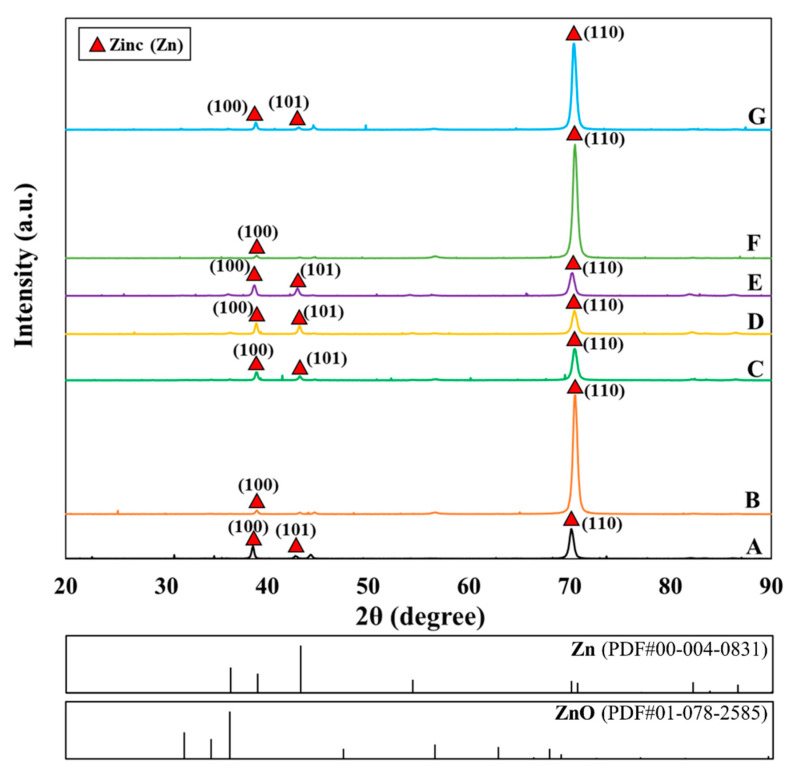
XRD profiles of specimens from different plating baths formulations.

The samples from different baths (A–G) were successfully prepared with good uniformity, bright appearance, and strong adhesion. The scanning electron micrographs obtained for all samples and a cross-sectional view of the representative sample from bath C are shown in Figure 2, which exhibit nanostructures, and their morphology can be classified largely into two types, according to their Zn and NaOH ratio: (i) Compact structure, as developed from baths A, B, C, F, and G, with a low-to-moderate Zn:NaOH ratio of 0.04–0.1, and (ii) porous dendritic structure, as developed from baths D and E, with a relatively high Zn:NaOH ratio above 0.1. Among the samples with compact morphology, samples from baths B and C show a woven structure comprising elongated rice-shaped grains, whereas others are composed of coalesced platelet crystallites with hexagonal shape. In terms of crystallographic orientation as assessed by XRD (Figure 3), the deposits are generally characterized by planes (100), (101), and (110). Strong preference of the (110) orientation is observed in some groups of the samples (B, F, and G). Some prior works have pointed to deposition conditions, including current density, plating bath formulation, and plating duration as factors influencing the morphology developments of electrogalvanized deposits [11,26,27]. Surface morphology of zinc coatings is found to play an important role controlling their corrosion properties [28,29].

### 3.1. Effects of Zinc Concentration

**Figure 4:**
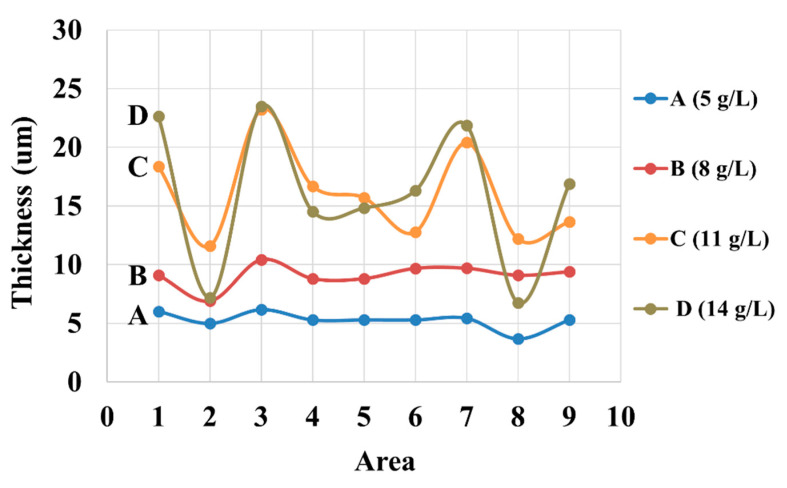
Thickness of coatings at different areas in the corrugated specimens of baths with different zinc concentrations. Three measurements were made for each sample and small standard deviations below 5% are observed for all data points.

Figure 4 shows the thickness of the Zn layer on the corrugated specimens obtained from baths A, B, C, and D. To put the coating thickness results in perspective, according to Faraday’s law, the electroplating of zinc with 100% cathodic current efficiency at 2 A/dm^2^ for 30 min will produce a coating layer with an average thickness of 17.1 µm. According to the thickness profiles in Figure 4, the overall thickness of the deposits increases with the Zn concentration in the baths. This could be due to the increment in the efficiency of zinc deposition as the concentration of electroactive ions increases [30,31]. On the other hand, the increase in Zn content appears to adversely affect the throwing power and uniformity of the coating. When the concentration of Zn is low (viz. baths A and B), the deposition rate, especially at the high-current-density area (e.g., edges or nongroove areas) is slow, resulting in a relatively more uniform coating thickness in all areas. For baths C and D, a high variation in coating thickness is present between the surface and groove areas. With a higher zinc concentration, the deposition rate is faster and is promoted preferentially on the surface as compared to the groove, leading to a reduced throwing power. Nevertheless, in practice, baths C and D could be favored over baths A and B due to a higher deposition rate.

Additionally, the results in Figure 4 indicate the hydrodynamic effect on the deposit thickness, especially for those with a high content of zinc in the electrolyte (bath C and D). Particularly, by considering the clockwise-rotating corrugated specimens, the electrode portion that moves toward the electrolyte (zone 1 and 3) tends to a have higher coating thickness, whereas the portion that moves away from the electrolyte (zone 7 and 9) shows a thinner deposit. This is because the local velocity of electrolyte adjacent to the cathode surface is fostered in the former case, resulting in the promotion of the rate of mass transport of the ions [32]. In the groove regions (zone 2 and 8), however, such an agitation effect is minimal and so is the hydrodynamic effect. 

**Figure 5:**
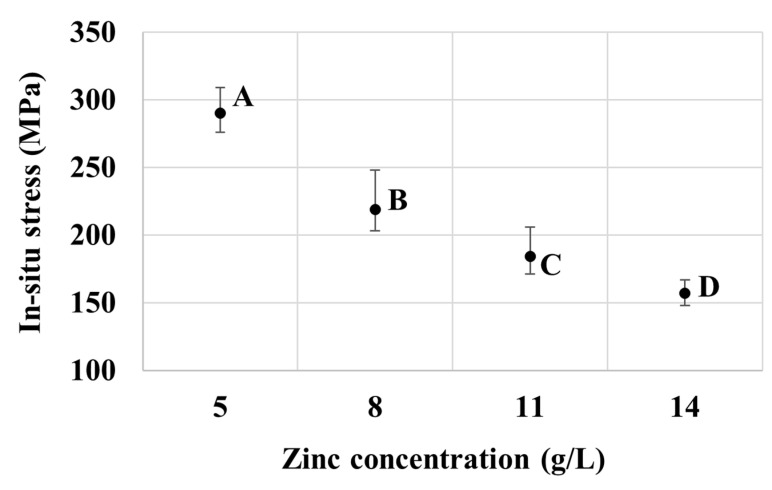
Effect of zinc concentration on the in-situ internal stress of samples from baths A, B, C, and D.

A closer inspection of the XRD profiles reveals a decreasing trend in the (110) peak intensity with the increasing bath concentration of zinc from bath B to C and D. From the higher surface density of (110) compared to (100), the change in surface morphology from a relatively dense state to a more porous nature of specimens from bath B to C and D in Figure 2 could be attributed to the decrease in the (110) texture. Figure 5 shows the internal stress of the deposit, which was developed during the electrogalvanizing session and measured in-situ for baths A to D. The stress is tensile and is reduced with the increasing Zn concentration in the plating baths. Particularly, samples from bath A of low Zn content (with a relatively thin coating layer) exhibit a higher tensile stress level than those with higher thickness, such as the sample obtained from bath D of high Zn content. This may be rationalized by the influence of crystallization and coating layer formations. It is known that tensile stresses could be established in the electrodeposits in the early stages, as grain boundary formation and crystallite coalescence proceed [30,33,34,35]. According to Hoffman et al. [33], tensile stress is generated due to the attractive force between adjacent grains that impinge on one another, as the grain boundaries are developed to form a continuous polycrystalline film. Abermann [35] reported that the degree of stress after crystallite coalescence depends largely on the mobility of the depositing atoms and the deposition rate. As the deposited film grows, tensile stresses could be relaxed, partly owing to an incorporation of atoms at the grain boundary and, therefore, accommodation or relaxation of stresses by the newly grown, relatively unstressed portion of the coating away from the interface between the substrate and the coating. Thus, this explains the relatively high tensile stress in the thin deposit from bath A, as opposed to the decreased tensile stress in the thick deposit from bath D.

**Figure 6:**
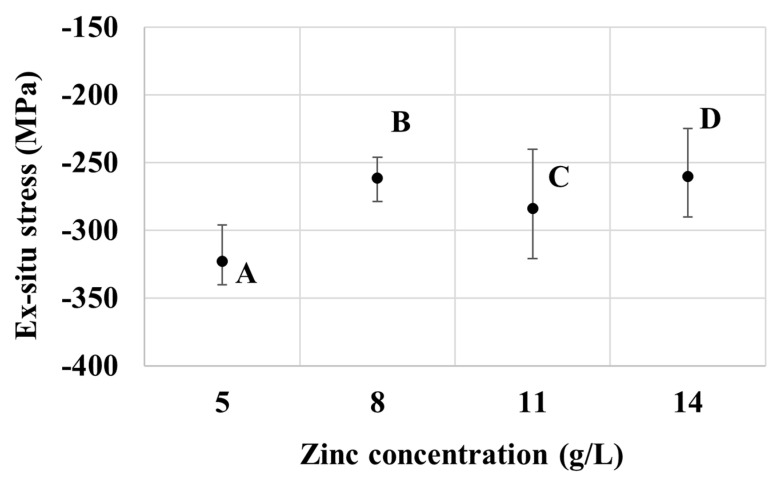
Effect of zinc concentration on the ex-situ stress of samples from baths A, B, C, and D.

Now, turning to the results from the ex-situ stress measurement presented in Figure 6, overall, the deposits from baths A to D exhibit compressive stresses. Furthermore, the samples from bath A appear to have a relatively high compressive stress magnitude compared to other sample groups. The compressive residual stress typically arises due to the incorporation of secondary atoms or molecules in the films, for example, at the triple junction or grain boundaries [36]. In this case, it is likely that hydrogen, which is primarily produced by the reduction reaction of water molecules at the cathode, is the key driver for stress development. Specifically, during a plating session, the generated hydrogen could adsorb and desorb from the surface of the working electrode at a certain rate according to thermodynamic equilibrium. However, once the plating session was finished and the sample was taken out of the plating bath, the desorption process was limited and the molecular hydrogen was mostly trapped in the deposit films [37,38]. Such occluded hydrogen could permeate to the grain boundaries, giving rise to the compressive stress in the deposits that could continue developing after the electrolytic part of the manufacturing process. This result in fact matches well with the common observation made by plating plants that blistering is one of the major defects of electrogalvanizing deposits, and that after each deposition session, additional annealing is recommended to release the post-plating compressive stresses [39]. This is also in line with the observed relative magnitude of compressive stresses in Figure 6. Particularly, the low zinc content in bath A would result in a low cathodic efficiency and a relatively high evolution rate of hydrogen instead, which could subsequently be adsorbed in the deposit films. The results in Figure 5 and Figure 6 therefore hint that the specimens from bath A, which have low zinc content and exhibit a relatively thin layer, are more prone to peeling-off during plating and to blistering after the plating session, as compared to other groups of specimens.

A consideration of the texture of the zinc deposits in Figure 3 also points to a possible contribution from the lattice mismatch between the steel substrate and the zinc deposit. The coatings produced from all bath formulations in the present work show a preferred prismatic (110) and (100) surface texture of zinc, indicating that these planes are oriented parallel to the coating surface, which implies that the (001) planes are oriented normal to the deposit surface. Therefore, lattice matching at the interface involves the basal plane of zinc, with the interplanar spacing d_Zn(001)_ of 4.947 Å. As the lattice constant of body-centered cubic iron is only 2.8655 Å, lattice accommodation between the film and the substrate would cause the steel substrate to be in tension, whereas the zinc film would be in compression. Given the larger contribution from the substrate, the length change of the overall substrate-film assembly would slightly increase during the deposition and, hence, the measured in-situ tensile internal stress. As the film thickens, the stress could be relaxed in the upper portion of the newly deposited film, resulting in a lower level of tensile internal stress from the length change method. The residual stress measured ex-situ using X-ray-based measurement would reflect the surface residual stress from the film and, hence, the compressive nature. As the film thickens, the stress measured at the surface would be lower as the measured region is further away from the interface between the substrate and the film, in line with the results in Figure 5 and Figure 6.

### 3.2. Effects of NaOH Concentration

**Figure 7:**
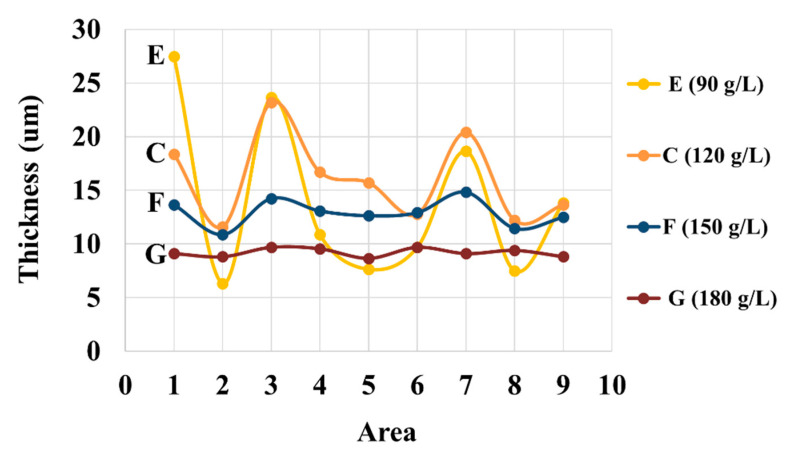
Thickness of coatings at different areas in the corrugated specimens of baths with different NaOH concentrations. Three measurements were made for each sample and small standard deviations below 5% are observed for all data points.

While zinc oxide is the source of zinc in the electrogalvanizing deposition from the alkaline non-cyanide bath, sodium hydroxide is generally responsible for improving electrolytic conductivity during the deposition, which in turn, promotes the coating thickness uniformity of the films [40]. Figure 7 shows the effect of the concentration of NaOH on the coating thickness of the corrugated specimens from bath C, E, F, and G. The high-NaOH specimens (bath G) exhibit a uniform but relatively thin coating. Further reduction in NaOH concentration from 180 to 150 g/L (bath F) appears to improve the cathodic efficiency, resulting in a much improved deposition rate, while maintaining good uniformity and leveling of coating on the surface and in the groove areas. High degrees of nonuniformity of the coatings are observed in the specimens from baths C and E with lower contents of NaOH. Furthermore, baths C and E experience the hydrodynamic effects more significantly, as compared to baths F and G. This is in line with the previous observation on the higher hydrodynamic effects induced in the baths with a high Zn-to-NaOH ratio. Regarding the effect on crystallographic orientation, with the constant zinc concentration of 11 g/L, an increase in the NaOH bath concentration can be correlated to an increase in the (110) peak intensity. Again, the increase in (110) orientation is found to accompany a denser surface morphology from specimens produced from bath E to C, F, and G.

**Figure 8:**
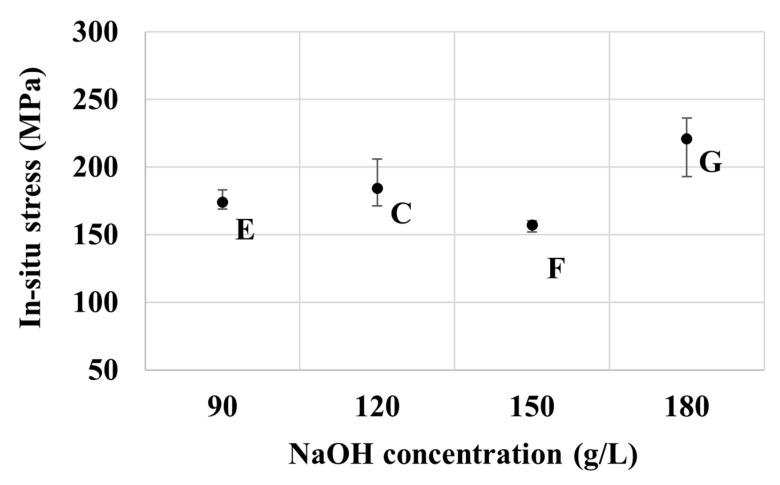
Effect of NaOH concentration on the in-situ internal stress of samples from baths E, C, F, and G.

The in-situ internal stress results of specimens from baths C, E, F, and G as presented in Figure 8 are generally correlated well to the measured thickness of the deposits, and hence, are in line with those observed from baths A–D. The relatively thick coatings as in those obtained from baths E and F exhibit relatively low tensile stress, whereas the relatively thin coating of bath G shows higher tensile stress. Likewise, the ex-situ stress results of specimens from baths C–G are also in the compressive regime, as observed previously from baths A–D. Interestingly, those from bath F exhibit the highest magnitude of compressive stress among the seven groups considered. This should be attributed to its relatively low in-situ tensile stress to start with, and the relatively high NaOH concentration that promotes the electrolysis reaction and, hence, the generation and occlusion of hydrogen at the electrode’s surface [41].

**Figure 9:**
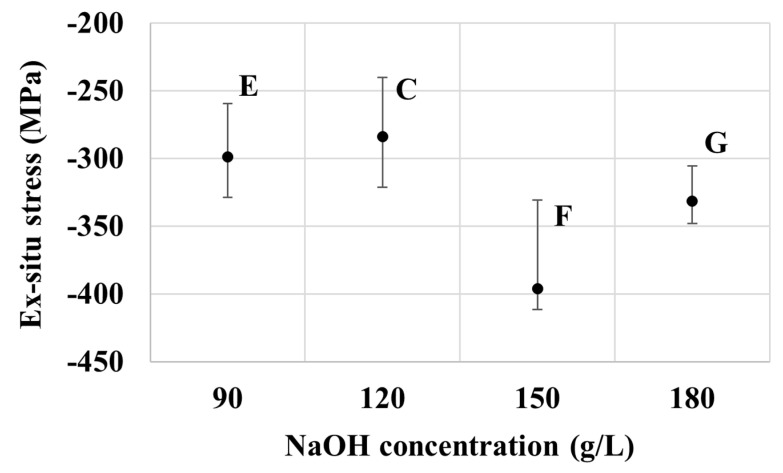
Effect of NaOH concentration on the ex-situ stress of the samples from baths E, C, F, and G.

Figure 8 and Figure 9 show the effect of NaOH concentration on in-situ and ex-situ stresses respectively. There is a good correlation in the trend of stress measured during and after the electroplating process. Overall, the tensile stress increases with the concentration of NaOH, except for at 150 g/L of NaOH, as seen in Figure 8. Deposits with thinner films have higher stresses. A similar trend of stress can be seen for the ex-situ stress in Figure 9. The compressive stress increased as the concentration of NaOH increased except for 150 g/L of NaOH. The mechanism explaining the development of tensile and compressive stress is as explained in the zinc part above.

## 4. Conclusions

A systematic study to analyze the effects of the concentration of Zn and NaOH on the plating characteristics in an alkaline non-cyanide zinc bath has been performed. Overall, the study demonstrates that bath formulations crucially control electrogalvanizing in many aspects, including coating surface morphology, deposition rate, throwing power, coating uniformity, and residual stress developments in the plating session and post-plating. The moderate Zn-to-NaOH ratio of about 0.067–0.092 (groups B, C, and F) appears to provide uniform and compact coatings, moderately high deposition rate, and relatively low residual stresses. The lower and higher Zn-to-NaOH ratios, on the other hand, generally lead to higher compressive stress after plating and the issues with coating buildups. Practically, electrogalvanizers therefore need to carefully maintain the bath formulation for optimum plating characteristics, especially for a long or continuous plating session as zinc is plated out steadily. In this regard, bath C could be favored over other formulations due to its larger processing window.

## Figures and Tables

**Table 1:** Experimental bath compositions.

	Zn (g/L)	NaOH (g/L)	Zn:NaOH Ratio
A	5	120	0.042
B	8	120	0.067
C	11	120	0.092
D	14	120	0.117
E	11	90	0.122
F	11	150	0.073
G	11	180	0.061
